# Acute kidney injury after CAR-T cell therapy: exploring clinical patterns, management, and outcomes

**DOI:** 10.1093/ckj/sfae123

**Published:** 2024-05-30

**Authors:** Maud Vincendeau, Adrien Joseph, Catherine Thieblemont, Florence Rabian, Stéphanie Harel, Sandrine Valade, Lara Zafrani

**Affiliations:** AP-HP, Hôpital Saint-Louis, Medical ICU, 1 avenue Claude Vellefaux, Paris, France; AP-HP, Hôpital Saint-Louis, Medical ICU, 1 avenue Claude Vellefaux, Paris, France; University Paris Cité, Paris, France; University Paris Cité, Paris, France; Hemato-oncology, DMU HI, AP-HP, Hôpital Saint-Louis, Research Unit NF-kappaB, Différenciation et Cancer, Paris, France; AP-HP, Hôpital Saint-Louis, Hematology Adolescent and Young Adults Unit, URP-3518, Paris, France; Immuno-Hematology Department, AP-HP, Hôpital Saint-Louis, Saint-Louis Hospital, Paris, France; AP-HP, Hôpital Saint-Louis, Medical ICU, 1 avenue Claude Vellefaux, Paris, France; AP-HP, Hôpital Saint-Louis, Medical ICU, 1 avenue Claude Vellefaux, Paris, France; University Paris Cité, Paris, France; INSERM UMR 944, University Paris Cité, Paris, France

**Keywords:** acute kidney injury, chimeric antigen receptor T cell, chronic kidney disease, cytokine release syndrome, intensive care unit

## Abstract

**Background:**

Acute kidney injury (AKI) has been reported after CAR-T cells, but available data are limited. We sought to describe the incidence of AKI in a cohort of patients hospitalized in the intensive care unit (ICU) following CAR-T cell reinjection, identify the primary factors linked to the onset of AKI, and ascertain the key determinants associated with kidney outcomes and mortality.

**Methods:**

We retrospectively analyzed 119 patients hospitalized in ICU after CAR-T cell therapy between 2017 and 2023. Factors associated with AKI, mortality, and kidney sequelae were identified using multivariate analyses.

**Results:**

Of the 119 patients, 41 patients fulfilled diagnostic criteria of AKI (34%). By multivariate analysis, grade ≥3 cytokine release syndrome (CRS) [OR = 1.20 CI95% (1.01–1.43)] and elevated lactate dehydrogenase (LDH) levels at admission [OR = 1.44 CI95% (1.04–1.99)] were significantly associated with the occurrence of AKI during ICU stay. AKI KDIGO ≥2 was an independent risk factor for hospital mortality [OR = 1.50 (1.22–1.85), *P *< 0.001]. Nine out of 12 (75%) and 6/9 (67%) patients who had experienced AKI and survived had chronic kidney disease (CKD) at 6 months and 1 year, respectively. We did not identify any specific factor associated with kidney recovery.

**Conclusion:**

AKI may occur in ICU patients receiving CAR-T cell therapy, especially those who experience CRS and exhibit elevated LDH levels. Early recognition of AKI is of utmost importance as it substantially compromises survival in these patients. Future studies should aim to elucidate the underlying pathophysiological mechanisms of AKI in this context and pinpoint predictive factors for long-term risks of CKD.

KEY LEARNING POINTS **What was known**:

- Limited data existed on acute kidney injury (AKI) in the context of CAR-T cell therapy.
- Previous studies lacked comprehensive analyses of urinary parameters in CAR-T recipients with AKI.
- A critical gap in the literature existed regarding AKI specifically within the intensive care unit (ICU) setting for CAR-T patients.

**This study adds**:

- Representation of one of the largest cohorts focusing specifically on AKI in CAR-T cell recipients admitted to the ICU.
- Details of complete urinary analyses, providing a comprehensive understanding of post-CAR-T therapy kidney function alterations.
- Status as the first study concentrating on AKI within the ICU setting, filling a critical gap in existing literature.

**Potential impact**:

- Enhances clinical practice by informing early recognition and intervention strategies for AKI in CAR-T recipients.
- Offers critical insights into the long-term outcomes, emphasizing the need for proactive management to mitigate chronic kidney disease among AKI survivors.

## INTRODUCTION

Since the late 2010s, the emergence of chimeric antigen receptor T cell (CAR-T) therapy has ushered in a transformative era in the management of specific hematological malignancies, yielding reductions in mortality rates and heralding prolonged periods of durable remission [1–4]. Initially approved for relapsed and refractory B cell acute lymphocytic leukemia in children and young adults [1, 5, 6], the scope of CAR-T applications has progressively broadened over time. Nevertheless, the efficacy of CAR-T cells is tempered by its inherent side effects; foremost of which is the cytokine release syndrome (CRS), which manifests within a window of 1 to 21 days post-infusion. Activation of CAR-T cells, on encountering their designated targets incites the liberation of effector cytokines, cascading into monocyte/macrophage system activation, and ensuing production of pro-inflammatory cytokines. This can precipitate a cascade of multi-visceral failure, encompassing acute kidney injury (AKI) as a prominent facet [7–9]. AKI may also be a consequence of severe sepsis [10, 11], or tumor lysis syndrome [12]. Rare cases of tubule-interstitial nephropathy have also been described [13].

Nonetheless, comprehensive insights into AKI due to CAR-T cells remain nascent. Data focusing on AKI are scarce and reported incidence of AKI following CAR-T cells infusion varies between 5% and 30% across diverse investigations, exhibiting varying degrees of association with mortality rates and prolonged stays within intensive care settings [7, 14, 15].

In this study, our primary objective was to identify the factors linked to the onset of AKI in a cohort of patients hospitalized in the intensive care unit (ICU) following CAR-T cell reinjection, while also delineating the incidence of AKI and ascertaining the key determinants associated with kidney outcomes and mortality.

## MATERIALS AND METHODS

### Patients

This retrospective study included all patients admitted in the ICU of Saint-Louis Hospital in Paris, following a CAR-T cell therapy session, between July 2017 and February 2023. Consecutive CAR-T cell recipients who required ICU admission within 30 days of CAR infusion were included.

Expanding on our center's prior studies about delayed ICU admission effects on critically ill immunocompromised patients, ICU admission was deemed appropriate in the following cases: sepsis requiring fluid resuscitation, acute respiratory failure with oxygen support ≥3 l/min, AKI, and new/high-risk organ dysfunction needing close monitoring.

### Data collection

The data in Tables 1–3 were abstracted from the medical records. Demographic parameters, medical history, presenting symptoms, and treatments were collected. All laboratory data were recorded at admission. Serum creatinine level was recorded within 3 months before admission and at admission, ICU discharge, 3 months, 6 months, 1 year, and last follow-up.

**Table 1:** Characteristics of the study population at ICU admission.

	No AKI	AKI	*P*
*n*	78	41	
Sociodemographic characteristics and comorbidities			
Age [mean (SD)]	56.60 (16.99)	52.86 (20.32)	0.290
Male sex (%)	42 (54.5)	32 (78.0)	0.021
Body mass index [mean (SD)]	24.0 (4.3)	25.1 (3.9)	0.165
Hypertension (%)	10 (13.0)	11 (26.8)	0.105
Congestive heart failure (%)	1 (1.3)	3 (7.3)	0.236
Diabetes (%)	3 (3.9)	4 (9.8)	0.382
Chronic liver disease (%)	1 (1.3)	1 (2.4)	1
CKD (%)	2 (2.6)	3 (7.3)	0.464
Baseline serum creatinine value [mean (SD)]	61 (21)	69 (23)	0.069
Performance status (%)			0.133
0	11 (14.3)	2 (4.9)	
1	29 (37.7)	23 (56.1)	
2	30 (39.0)	11 (26.8)	
3	7 (9.1)	5 (12.2)	
Hematological disease			0.797
Acute lymphoblastic leukemia	13 (16.9)	9 (22.0)	
Diffuse large B cell lymphoma	62 (80.5)	31 (75.6)	
Myeloma	2 (2.6)	1 (2.4)	
Number of previous therapeutic lines [mean (SD)]	3.05 (1.16)	3.56 (1.69)	0.056
Autologous stem cell transplant (%)	15 (19.5)	4 (9.8)	0.269
Allogenic stem cell transplant (%)	3 (3.9)	3 (7.3)	0.715
Disease status before lymphodepletion (%)			0.492
Stable disease	10 (13)	2 (4.9)	
Progression	44 (57.1)	24 (58.5)	
Relapse	15 (19.5)	9 (22.0)	
Complete response	2 (2.6)	0 (0.0)	
Partial response	6 (7.8)	6 (14.6)	
CAR-T cell (%)			0.802
Brexucabtagene autoleucel (Tecartus®)	4 (5.4)	3 (7.3)	
Tisagenlecleucel (Kymriah®)	29 (39.2)	15 (36.6)	
Axicabtagene ciloleucel (Yescarta®)	37 (50.0)	19 (46.3)	
Other	4 (5.4)	4 (9.8)	
At ICU admission			
Time between hospital and ICU admission (days) [mean (SD)]	14.56 (9.21)	15.85 (9.98)	0.481
Time between CAR-T cell infusion and ICU admission (days) [mean (SD)]	6.04 (3.06)	6.46 (4.70)	0.555
Reason for admission (%)			0.012
Hemodynamic failure	36 (46.8)	25 (61.0)	
Neurological failure	26 (33.8)	3 (7.3)	
Kidney/metabolic failure	0 (0.0)	1 (2.4)	
Respiratory failure	4 (5.2)	6 (14.6)	
Close monitoring	9 (11.7)	6 (14.6)	
Other	2 (2.6)	0 (0.0)	
Infection at ICU admission (%)			0.097
No	45 (59.2)	19 (46.3)	0.2545
Bacterial	29 (38.2)	20 (48.8)	0.360
Viral	2 (2.6)	0 (0.0)	0.764
Fungal	0 (0.0)	5 (9.8)	0.024
SOFA score [mean (SD)]	3.83 (2.05)	6.00 (4.07)	<0.001
SAPSII score [mean (SD)]	41.62 (13.06)	47.03 (17.02)	0.129
Temperature (°C) [mean (SD)]	39.25 (1.14)	39.05 (1.31)	0.414
Mean blood pressure (mmHg) [mean (SD)]	69.27 (13.36)	67.60 (16.59)	0.560
Blood lactate level (mmol/l) [mean (SD)]	1.57 (1.02)	1.95 (1.75)	0.256
Urea (mmol/l) [mean (SD)]	3.99 (1.75)	6.14 (4.12)	<0.001
Serum creatinine (µmol/L) [mean (SD)]	63.99 (24.01)	111.07 (77.17)	<0.001
LDH (IU/L) [mean (SD)]	430.03 (357.88)	1231.17 (2647.41)	0.010
Troponine level (ng/ml) [mean (SD)]	18.23 (16.66)	38.18 (40.88)	0.014
Leucocytes (×10^3^/l) [mean (SD)]	1837.01 (2879.35)	1324.39 (2114.82)	0.317
Neutrophil (×10^3^/l) [mean (SD)]	2475.71 (1984.72)	1975.00 (2354.67)	0.769
CRS grade on ICU admission (%)			0.025
0	10 (13.0)	7 (17.1)	
1	31 (40.3)	8 (19.5)	
2	33 (42.9)	19 (46.3)	
3	3 (3.9)	7 (17.1)	

**Table 2:** Characteristics of the study population during ICU stay.

During ICU stay	No AKI	AKI	*P*
*n*	78	41	
Worst CRS grade during ICU stay (%)			0.012
0	4 (5.3)	4 (9.8)	
1	30 (39.5)	7 (17.1)	
2	32 (42.1)	15 (36.6)	
3	10 (13.2)	14 (34.1)	
4	0 (0.0)	1 (2.4)	
Respiratory failure (%)	29 (37.7)	29 (70.7)	0.001
Disseminated intravascular coagulation (%)	9 (11.7)	11 (26.8)	0.067
Neurological toxicity (ICANS) (%)	36 (46.8)	20 (48.8)	0.987
Worst ICANS grade (%)			0.829
0	42 (54.5)	20 (48.8)	
1	9 (11.7)	5 (12.2)	
2	7 (9.1)	3 (7.3)	
3	7 (9.1)	3 (7.3)	
4	12 (15.6)	10 (24.4)	
Antibiotics during the first 3 days after ICU admission (%)	76 (98.7)	40 (97.6)	1
Infection during ICU stay (%)			0.472
No infection	50 (64.9)	24 (58.5)	
Lung infection	2 (2.6)	3 (7.3)	
Urinary tract infection	4 (5.2)	2 (4.9)	
Digestive tract	2 (2.6)	3 (7.3)	
Cutaneous	2 (2.6)	2 (4.9)	
Catheter	16 (20.8)	5 (12.2)	
Primary bacteriemia	1 (1.3)	1 (2.4)	
Other	0 (0.0)	1 (2.4)	
Proven fungal infection (%)	0 (0.0)	5 (9.8)	0.024

**Table 3:** Characteristics of AKI.

*n*	41
At ICU admission	
Serum	
Urea (mmol/l) [mean (SD)]	6.14 (4.12)
Creatinine (µmol/l) [mean (SD)]	111.07 (77.17)
Potassium (mmol/l) [mean (SD)]	3.70 (0.50)
Phosphorus (mmol/l) [mean (SD)]	0.90 (0.31)
Calcium (mmol/l) [mean (SD)]	2.09 (0.17)
Uricemia (µmol/l) [mean (SD)]	233.89 (163.01)
LDH (IUI/l) [mean (SD)]	878.81 (1500.41)
Peak serum creatinine (µmol/l) [mean (SD)]	166 (135)
Day of peak serum creatinine [mean (SD)]	3.36 (3.46)
KDIGO stage (%)	
1	25 (61.0)
2	6 (14.6)
3	10 (24.4)
Urine	
Sodium (mmol/l) [mean (SD)]	82.34 (52.01)
Potassium (mmol/l) [mean (SD)]	36.48 (21.62)
Urea (mmol/l) [mean (SD)]	165.82 (101.28)
Creatinine (mmol/l) [mean (SD)]	6.17 (3.48)
Protein (g/d) [mean (SD)]	0.46 (0.49)
Protein/creatinine ratio [mean (SD)]	60.27 (42.99)
Hematuria (%)	11 (27.5)
Leucocyturia (%)	10 (25.0)
Fractional excretion of sodium [mean (SD)]	1.59 (1.69)
Fractional excretion of urea [mean (SD)]	46.52 (13.76)
Outcomes	
RRT (%)	3 (7.3)
Serum creatinine at ICU discharge (µmol/l) [mean (SD)]	103.17 (84.00)
Death (%)	
in the ICU	3 (7.7)
in the hospital	8 (22.9)
at 3 months	14 (37.8)
at 6 months	17 (47.2)
Serum creatinine at 6 months (µmol/l) [mean (SD)]	98.32 (47.79)
Serum creatinine at 12 months (µmol/l) [mean (SD)]	95.00 (46.82)

Vital status at ICU discharge, hospital discharge, at 6 months and 1 year were determined from medical records and the outpatient clinic electronic database.

### Definitions

Definition and staging of AKI were defined according to the 2012 KDIGO (Kidney Disease: Improving Global Outcomes) guidelines [16]. The creatinine value used for baseline was the value obtained 3 months before ICU admission.

Definition and staging for CKD (chronic kidney disease) were defined according to the 2012 KDIGO guidelines (including both creatinine levels and urine output criteria) [17].

Proteinuria was defined as protein-creatinine ratio >30 mg/mmol or protein excretion >0.3 g/d.

Decisions regarding the initiation, discontinuation, and modalities of renal replacement therapy (RRT) were made by the physician in charge of the patient.

Recovery of kidney function was defined as a reduction in peak AKI stage based on KDIGO criteria at hospital discharge [18]. If hemodialysis treatment was discontinued and kidney function returned to baseline, recovery of kidney function was considered complete.

Patients were considered to have ‘kidney sequelae’ if they did not achieve recovery of kidney function (i.e. reduction in peak AKI stage based on KDIGO criteria) at hospital discharge.

CRS grading and immune effector cell-associated neurotoxicity syndrome (ICANS) grading were based on the ASBMT classification [19].

The Sepsis-Related Organ Failure Assessment (SOFA) score was calculated at ICU admission as previously defined [20].

### Outcomes

The primary objective of the study was to identify risk factors associated with AKI in CAR-T cell recipients. Secondary objectives were to decipher mechanisms of AKI in CAR-T cell recipients, to identify factors associated with kidney outcomes and mortality in AKI patients.

### Statistical analysis

Continuous data were described as mean and standard deviation and categorical data are presented as numbers and percentages of total. Comparison of characteristics between patients with and without AKI was made using a Student’s *t*-test or Fisher’s exact test, as appropriate. Variables significantly associated with AKI or hospital survival in univariate analysis were included in multivariate logistic regression models after selection of clinically relevant variables. Kaplan–Meier curves until 1 year after ICU admission were stratified according to AKI and compared using a log-rank test. *P* values <0.05 were considered statistically significant, and all statistical tests were two-sided. Statistical analyses were performed using R version 3.4.2 (R Foundation for Statistical Computing, Vienna, Austria; https://www.R-project.org/).

### Ethic approval

This study was approved by a local ethic committee (Société de Réanimation de Langue Française, CE SRLF 19–04). According to French law, need for informed consent was waived. This is in adherence to the Declaration of Helsinki.

## RESULTS

### Patient characteristics

**Figure 1:**
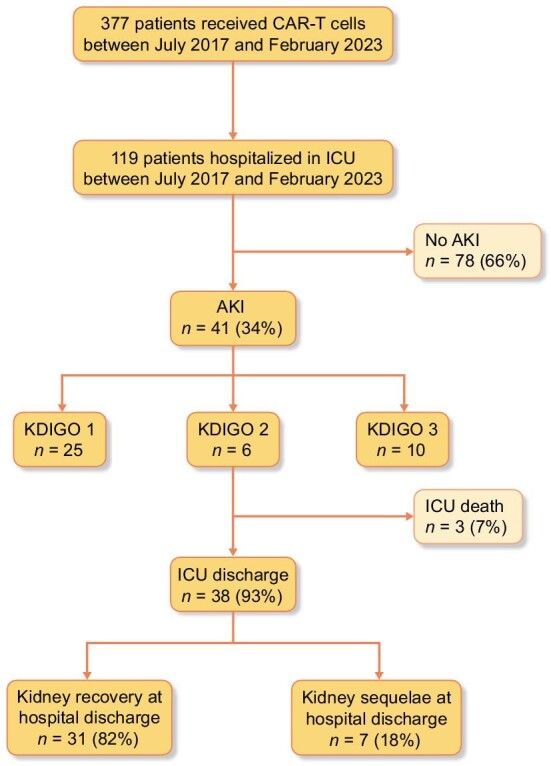
Patient flow chart.

Three hundred and seventy-seven patients received CAR-T cell therapy in the study period in the different hematology departments of Saint-Louis hospital. One hundred and nineteen patients with a mean age of 55 years (±18) were hospitalized in ICU and included (Fig. 1). Patient characteristics at ICU admission are reported in Table 1. The underlying hematological malignancy was Diffuse Large B Cell Lymphoma in 93 patients (75.6%), acute lymphoblastic leukemia in 22 patients (22%), and myeloma in three patients (2.5%). Patients underwent a mean of three lines of chemotherapy before CAR-T cell therapy. All patients had received conditioning chemotherapy with cyclophosphamide and fludarabine before CAR-T cell infusion. Autologous CAR-T cells were used in all patients except one who received allogenic CAR-T cells. Concerning CAR-T cells, axicabtagene ciloleucel was administered in 56 (47%) patients, while tisagenlecleucel was given to 44 (37%) patients and brexucabtagene autoleucel to 7 (6%) patients (Table 1).

**Figure 2:**
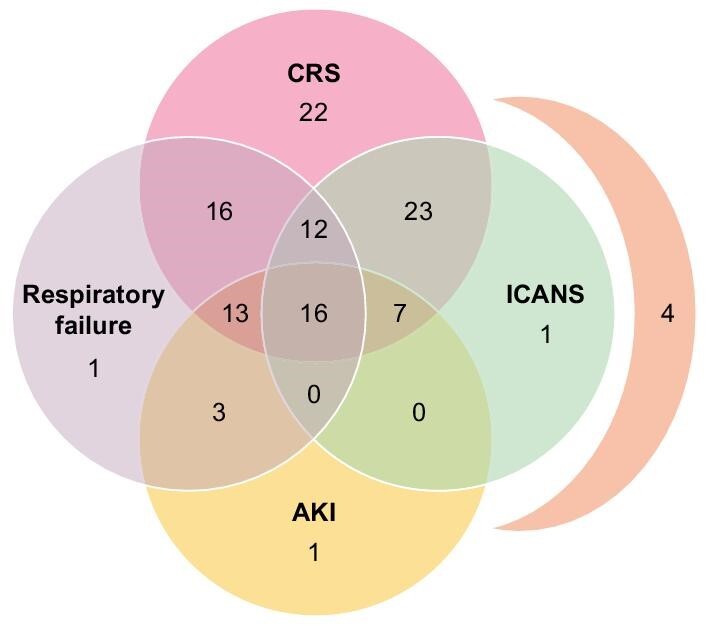
Clinical presentation and CAR-T related toxicities in the study population. The group of four patients corresponds to patients who experienced CRS and AKI, without ICANS.

The mean time between injection of CAR-T cells and ICU admission was 6 days. More than half of patients had grade 2–3 CRS on admission. Isolated CRS (*n* = 22, 18.6%), and sepsis with bacterial documented infections (*n* = 20, 16.8%) were the most common CAR-T cell-related complications. ICANS occurred in 56 (47%) patients (Table 2, Fig. 2).

### Characteristics of AKI, risk factors for AKIm and kidney outcomes

During the study period, 41 patients fulfilled diagnostic criteria of AKI (34%). Among these, 25 patients (61%) had AKI KDIGO 1, six patients (15%) KDIGO 2, and 10 patients (24%) KDIGO 3 (Table 3). Three (7%) patients required RRT. In the AKI group, patients were mostly admitted because of hemodynamic failure (*n* = 25, 61%). Twenty-nine patients (71%) had concomitant respiratory failure and 20 patients (49%) had neurological disorders at ICU admission. Six patients (14.6%) were placed on mechanical ventilation. Vasopressor support was used in 19 patients (46.3%) (Table 2). ICU mortality rate in the AKI group was 7.7% (*n* = 3). Mortality rate in AKI patients was 47% (*n* = 17) at 6 months.

Characteristics of AKI are described in Table 3. Urinary analyses were available in 38 patients (93%): 11 patients (27.5%) had significant hematuria and 10 patients (25%) had aseptic leukocyturia. Among them, quantitative polymerase chain reaction was performed in urine of two patients to measure CAR transgene expression and found 100 copies of transgene DNA/µg human genomic DNA for the first patient and 58 copies of transgene DNA/µg human genomic DNA for the second patient.

Proteinuria was low, with a mean proteinuria/creatinuria score of 60.3 mg/mmol (SD 42.99) without significant albuminuria.

Urinary ionograms supported an organic cause of AKI (urea excreted fraction >35%) in five (18%) patients. No AKI resulted from obstructive uropathy.

A kidney biopsy in one patient revealed acute tubular necrosis and a small CD19-negative interstitial infiltrate, ruling out infiltration by CAR-T cells.

No patient experienced tumor lysis syndrome in our cohort.

**Figure 3:**
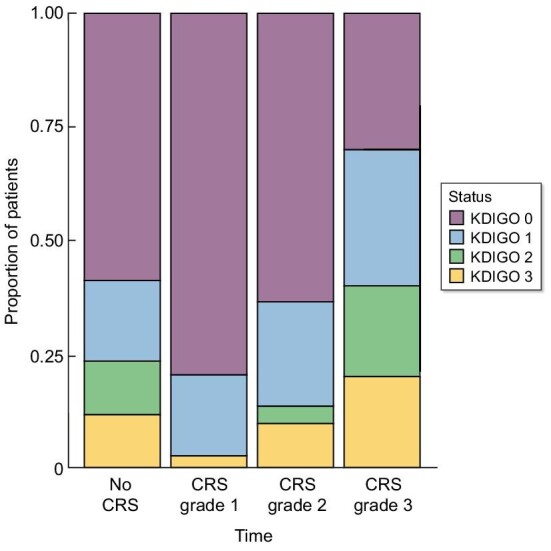
Severity of AKI according to CRS grade (worst CRS grade during ICU stay).

The grade of CRS was associated with the severity of AKI (*P *< 0.001), with 25% of patients with grade 3 CRS presenting with KDIGO 3 AKI (Fig. 3).

**Figure 4:**
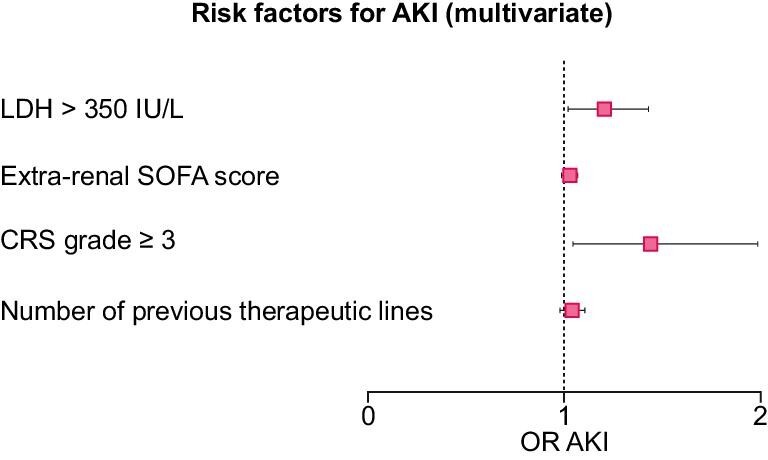
Forest plot distribution of the factors associated with AKI (multivariate).

By multivariate analysis, grade ≥3 CRS [OR = 1.44 CI95% (1.04–1.99)] and a lactate dehydrogenase (LDH) level on admission greater than 350 IU/L [OR = 1.20 CI95% (1.01–1.43)] were significantly associated with the occurrence of AKI during ICU stay (Fig. 4 and Supplemental Fig. S1).

**Figure 5:**
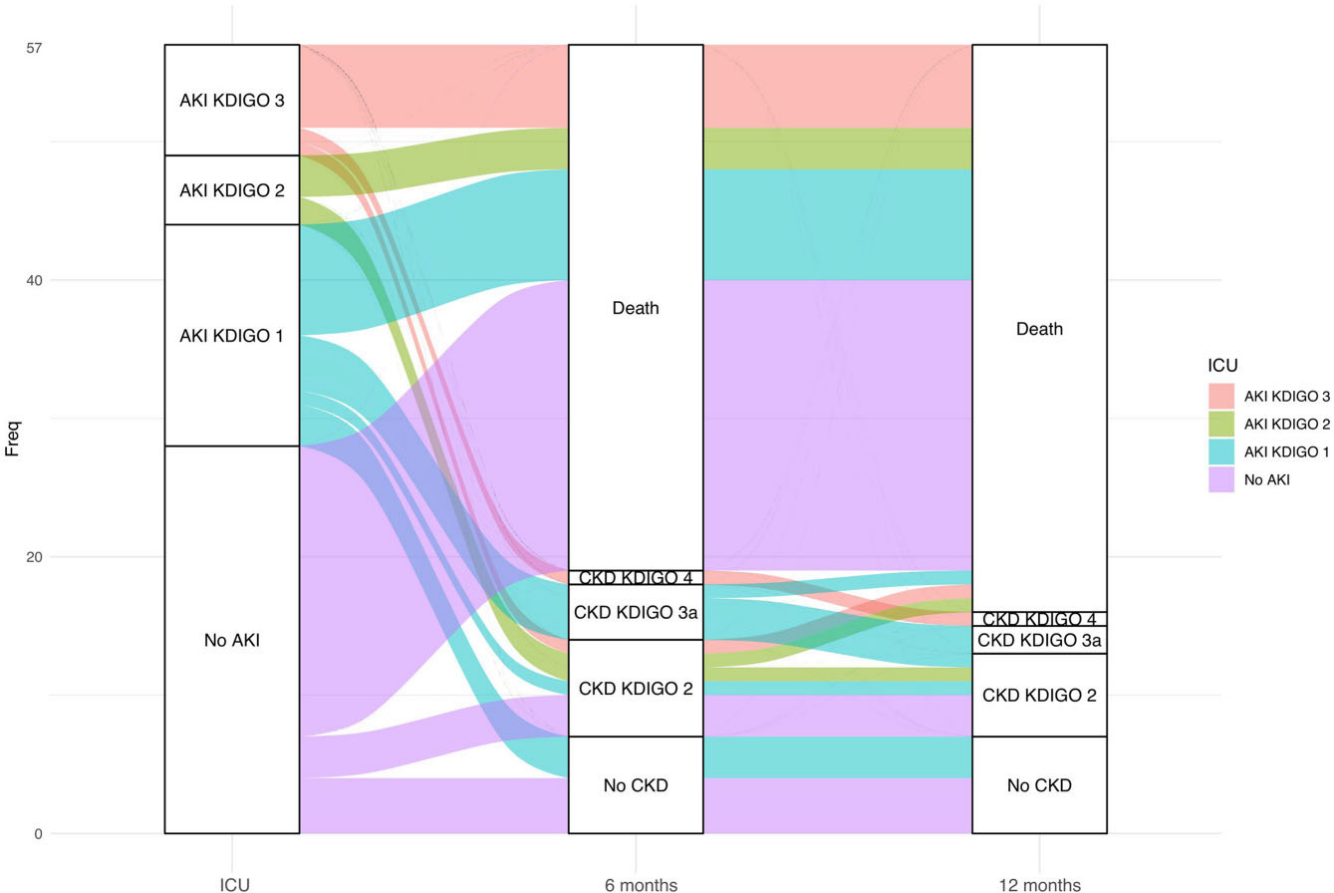
Evolution of kidney function in AKI patients 6 months and 1 year after ICU stay. Only survivors at ICU discharge are considered.

Only 82% of survivors had kidney recovery at discharge from the hospital. 9/12 (75%) and 6/9 (67%) patients who had experienced AKI and survived had CKD at 6 months and 1 year, respectively (Fig. 5). No factor was significantly associated with kidney recovery (Supplemental Table S1).

### Impact of AKI on mortality

**Figure 6:**
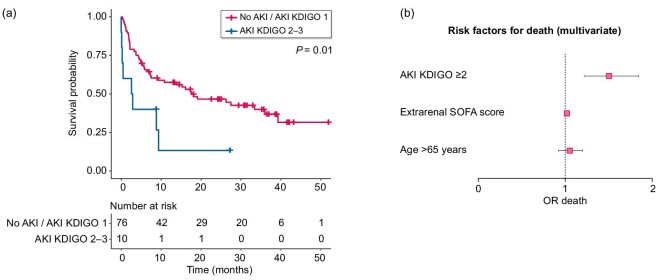
Severe AKI is associated with mortality. (**a**) Kaplan–Meier survival estimates according to the presence or not of severe AKI (KDIGO 2–3). (**b**) Forest plot distribution of the factors associated with hospital mortality (multivariate).

Mortality was significantly higher in the AKI KDIGO 2 or 3 group when compared with the no AKI or AKI KDIGO 1 group (Fig. 6a). Univariate analysis of factors associated with mortality is shown in Supplemental Table S2.

By multivariable analysis, AKI KDIGO 2 or more remained an independent risk factor for hospital mortality [OR = 1.50 (1.22–1.85), *P *< 0.001] (Fig. 6b).

In terms of the influence of targeted treatments for CRS on mortality and/or kidney sequelae, we did not found a statistically significant association between any specific treatment and mortality/kidney sequelae, although there was a trend favoring Tocilizumab [OR = 0.71 (0.51–0.99), *P *= 0.053], in contrast with siltuximab [OR = 0.98 (0.66–1.44), *P *= 0.901] or steroids [OR = 1.04 (0.73–1.49), *P *= 0.809].

## DISCUSSION

CAR-T cell therapy, while representing a significant advancement in treating refractory hematological malignancies, is also increasingly recognized as a cause of AKI. This cohort is one of the largest cohorts focusing on AKI in CAR-T cell recipients, the first to describe complete urinary analyses in these patients and the first to focus on AKI in the ICU setting. We observed an incidence of AKI of 34% in our cohort, which is higher than previously reported [8, 15]. In Kanduri *et al*.’s meta-analysis of articles reporting the incidence of AKI among patients receiving CAR-T therapies, AKI was reported in 19% of CAR-T cells recipients, with full recovery of kidney function in most cases (15). Other studies have reported incidences ranging from 5% to 30% [7, 8, 21]. These variations can be partially attributed to differences in the AKI definitions used across studies. We chose to adopt KDIGO definitions, known for their sensitivity in detecting even subtle changes in kidney function. Additionally, given our focus on critically ill patients, our cohort is more susceptible to AKI occurrences.

The association of severe AKI (KDIGO 2 and 3) with mortality is consistent with the existing literature. Indeed, in Gupta *et al*.’s study, 15 patients (among 78 CAR-T recipients) experienced AKI, those with acute tubular necrosis and obstruction exhibited the highest 60-day mortality rate [7]. Similarly, Wood *et al.*, who investigated the outcomes of CAR-T recipients, including those with pre-existing CKD, also reported an association between AKI and elevated mortality rates [22]. Therefore, AKI emerges as a significant factor to consider when evaluating the prognosis of patients undergoing CAR-T cell therapy.

We found that CRS and LDH levels were the main factors associated with the occurrence of AKI. Moreover, the grade of CRS was correlated with the severity of AKI. Similarly, Myers *et al.* showed that patients with high-grade CRS exhibited a fivefold increase in the risk of developing AKI [23]. Additionally, Gupta *et al.* observed that patients with severe AKI had more severe CRS, further emphasizing the association between high-grade CRS and AKI [7]. The mechanisms underlying AKI following CRS may involve several intricate pathways. The cytokine cascade can lead to increased vascular permeability third-spacing, hypotension and subsequent renal hypoperfusion. However, we found that CRS was significantly associated with AKI in multivariate analysis, independently of the extra-renal SOFA score, which encompasses parameters related to hemodynamic status: this suggests that additional mechanisms may be involved in the development of AKI. Several cytokines, including Interleukin-6 (IL-6), soluble IL-6 receptor, Interferon-γ (IFN- γ), and IL-8, are highly elevated during CRS [24, 25]. These cytokines may cause direct tubular injury or kidney inflammation [26]. Moreover, IL-6 has been demonstrated to play a crucial role in glomerular inflammation in various glomerular diseases [27]. However, in our cohort, we did not detect significant proteinuria that would suggest an underlying glomerular injury. Acharya *et al.* also documented a case of collapsing glomerulopathy following CAR-T administration [28]. This case lends support to the notion that cytokines may contribute to the initiation or exacerbation of collapsing glomerulopathies, particularly in genetically predisposed patients.

An interesting finding in our study is also the significant association between LDH levels and the occurrence of AKI, independently of the presence of CRS. LDH may be considered as a surrogate marker of tumor burden in these patients who may have kidney infiltration of tumor cells, especially in lymphoma patients with high tumor burden [29]. AKI secondary to interstitial T cell infiltration have also been described in several case reports, especially in kidney transplant recipients having received CAR-T cell therapy for post-transplant B cell lymphoma [13, 30]. However, T cell infiltration in these cases did not correspond to CAR-T infiltration, but primarily involved endogenous CD19 negative T lymphocytes. Notably, aseptic leucocyturia and microscopic hematuria were found in 10 (25%) of AKI patients within our cohort. Interestingly, two of these patients were identified as having CAR-T cells present in their urine analysis, as confirmed by PCR detection of the CAR-T transgene. Whether these patients have CAR-T infiltration in the kidney parenchyma and secondary tubulointerstitial nephritis is unknown. Unfortunately, only one patient in our cohort underwent kidney biopsy for persistent AKI that showed acute tubular necrosis. The limited frequency of kidney biopsies can be attributed to physicians' apprehensions regarding the heightened risk of bleeding complications in patients with thrombocytopenia and/or bleeding disorders, a scenario frequently encountered in the weeks following chemotherapy for lymphodepletion, which is typically performed the week preceding CAR-T cells infusion.

No patient experienced tumor lysis syndrome after CAR-T infusion within the entire cohort. Tumor lysis syndrome has been reported in few case reports in patients receiving CAR-T cell for refractory chronic lymphocytic leukemia [12], within an onset occurring 2 to 3 weeks after CAR-T infusion. The exact incidence of AKI-induced tumor lysis syndrome remains unknown in this context but appears to be quite rare. We can postulate that the gradual eradication of tumor cells by CAR-T cells likely provides partial protection against tumor lysis syndrome.

Only three patients have been treated by CAR-T cell for myeloma in our cohort. CAR-T cell therapy in myeloma patients was initiated in our center in 2020, which may explain the low number of myeloma patients with AKI. None of these patients had a light chain cast nephropathy when they received CAR-T cells. This will need further investigation in the future, especially in patient who have myeloma cast nephropathy. Nevertheless, no episodes of AKI secondary have been reported by Raje *et al.* who studied the initial toxicity profile of anti-B Cell Maturation Antigen CAR-T for patients with relapsed or refractory multiple myeloma [4].

Regarding targeted treatments for CRS, we did not find a statistically significant association between any specific treatment and mortality, although there was a trend favoring Tocilizumab alone when compared to patients who had received corticosteroids and siltuximab. However, as corticosteroids and siltuximab are most often used as second line therapies, this trend may reflect the severity of the patients rather than a causal effect of Tocilizumab on mortality and kidney outcomes.

Among patients who experienced AKI during ICU stay, a significant proportion of survivors had kidney sequelae with CKD ranging from grade 2 to grade 4, underlying the importance of careful clinical–biological follow-up and the need for specialized long-term follow-up by a nephrology team. Unfortunately, our study lacked the statistical power required to pinpoint specific factors associated with kidney recovery.

While our retrospective analysis provides valuable insights into the incidence and outcomes of AKI in patients receiving CAR-T cell therapy, several limitations should be acknowledged. First, the single-center nature of our study may restrict the generalizability of our findings to broader populations. Variations in patient demographics, treatment protocols, and healthcare practices across different centers may influence the prevalence and outcomes of AKI in CAR-T cell therapy recipients. Additionally, the retrospective design inherently introduces biases and limitations inherent to data collection and analysis. Finally, the number of patients who developed AKI in our cohort was relatively small, which may limit the statistical power to detect significant associations.

In conclusion, AKI is common in patients undergoing CAR-T cell therapy, especially those requiring ICU admission, those who experience CRS and exhibit elevated LDH levels. Early recognition of AKI is of utmost importance as it substantially compromises survival in these patients. Future research should focus on unraveling the intricate pathophysiological mechanisms of AKI in this context and pinpoint predictive factors for long-term risks of CKD.

## Supplementary Material

sfae123_Supplemental_File

## Data Availability

Study protocol, data set, and statistical code are available from the corresponding author on reasonable request.
