# Supplementary material for: Acute kidney injury after CAR-T cell therapy: exploring clinical patterns, management, and outcomes
Source: Clin Kidney J. 2024 May 30;17(6):sfae123. doi: 10.1093/ckj/sfae123 (PMC11195623; doi:10.1093/ckj/sfae123)
Supplement: sfae123_Supplemental_File [file sfae123_supplemental_file.docx]

**Supplemental data**

**Supplemental Table S1 : Factors associated with kidney recovery at discharge**

|  | Odd Ratio | p |
| --- | --- | --- |
| Age | 1.002 [0.995 - 1.008] | 0.612 |
| Male sex | 1.284 [0.961 - 1.716] | 0.010 |
| Hemodynamic failure | 0.914 [0.709 - 1.178] | 0.490 |
| Body mass index | 0.998 [0.966 - 1.03] | 0.881 |
| SOFA score | 1.012 [0.982 - 1.044] | 0.446 |
| Prothrombin time | 1.004 [0.995 - 1.013] | 0.375 |
| Cytokine release syndrome (CRS) | 0.941 [0.619 - 1.43] | 0.777 |
| Grade 3 CRS | 1.325 [0.964 - 1.822] | 0.091 |
| Respiratory failure | 0.925 [0.705 - 1.215] | 0.580 |
| Neurological failure | 1.227 [0.965 - 1.561] | 0.103 |
| Mechanical ventilation | 0.967 [0.68 - 1.375] | 0.854 |
| Vasopressor support | 1.252 [0.986 - 1.591] | 0.073 |
| Documented infection | 1.03 [0.792 - 1.338] | 0.829 |
| Tocilizumab | 0.957 [0.708 - 1.295] | 0.779 |
| Siltuximab | 1.126 [0.811 - 1.565] | 0.483 |
| Corticosteroids | 1.13 [0.846 - 1.509] | 0.413 |

**Supplemental Table S2. Univariate analysis of factors associated with mortality**

|  | Alive at hospital discharge | Hospital death | p |
| --- | --- | --- | --- |
| n | 77 | 12 |  |
| **Sociodemographic characteristics and comorbidities** |  |  |  |
| Age (mean (SD)) | 52.95 (19.50) | 56.62 (19.07) | 0,544 |
| Male sex (%) | 49 (63.6) | 9 (75.0) | 0,658 |
| Weight (mean (SD)) | 72.53 (14.09) | 76.88 (15.07) | 0,328 |
| Hypertension (%) | 10 (13.0) | 6 (50.0) | 0,007 |
| Congestive heart failure (%) | 3 (3.9) | 1 (8.3) | 1 |
| Diabetes (%) | 5 (6.5) | 1 (8.3) | 1 |
| Chronic liver disease (%) | 2 (2.6) | 0 (0.0) | 1 |
| Chronic kidney disease (%) | 2 (2.6) | 1 (8.3) | 0,87 |
| Baseline serum creatinine value (mean (SD)) | 63.79 (23.31) | 60.75 (21.71) | 0.673 |
| Performance status (%) |  |  | 0,026 |
| 0 | 13 (16.9) | 0 (0.0) |  |
| 1 | 40 (51.9) | 3 (25.0) |  |
| 2 | 17 (22.1) | 7 (58.3) |  |
| 3 | 7 (9.1) | 2 (16.7) |  |
| Hematological disease |  |  | 0.905 |
| Acute lymphoblastic leukemia | 17 (22.1) | 3 (25.0) |  |
| Diffuse large B cell lymphoma | 59 (76.6) | 9 (75.0) |  |
| Myeloma | 1 (1.3) | 0 (0.0) |  |
| Number of previous therapeutic lines (mean (SD)) | 3.42 (1.34) | 3.83 (1.70) | 0.336 |
| Autologous stem cell tranplant (%) | 12 (15.6) | 0 (0.0) | 0,31 |
| Allogenic stem cell tranplant (%) | 4 (5.2) | 1 (8.3) | 1 |
| Disease status before lymphodepletion (%) |  |  | 0,87 |
| Stable disease | 9 (11.7) | 2 (16.7) |  |
| Progression | 43 (55.8) | 7 (58.3) |  |
| Relapse | 19 (24.7) | 3 (25.0) |  |
| Complete response | 1 (1.3) | 0 (0.0) |  |
| Partial response | 5 (6.5) | 0 (0.0) |  |
| CAR T cell (%) |  |  | NaN |
| Brexucabtagene Autoleucel (Tecartus®) | 6 | 0 |  |
| Tisagenlecleucel (Kymriah®) | 34 | 3 |  |
| Axicabtagene ciloleucel (Yescarta ®) | 34 | 8 |  |
| Other | 3 | 1 |  |
| **At ICU admission** |  |  |  |
| Time between hospital and ICU admission (days) (mean (SD)) | 14.25 (8.02) | 21.50 (11.02) | 0,007 |
| Reason for admission (%) |  |  | NaN |
| Hemodynamic failure | 42 (54.5) | 6 (50.0) |  |
| Neurological failure | 15 (19.5) | 3 (25.0) |  |
| Kidney / Metabolic failure | 0 (0.0) | 0 (0.0) |  |
| Respiratory failure | 9 (11.7) | 0 (0.0) |  |
| Close monitoring | 9 (11.7) | 3 (25.0) |  |
| Other | 2 (2.6) | 0 (0.0) |  |
| Infection at ICU admission (%) |  |  | 0,003 |
| No | 42 (54.5) | 4 (33.3) |  |
| Bacterial | 35 (45.5) | 6 (50.0) |  |
| Viral | 0 (0.0) | 1 (8.3) |  |
| Fungal | 0 (0.0) | 1 (8.3) |  |
| SOFA score (mean (SD)) | 4.12 (2.69) | 7.00 (5.06) | 0,003 |
| SAPSII score (mean (SD)) | 42.40 (13.41) | 53.67 (22.33) | 0,035 |
| Temperature (°C) (mean (SD)) | 39.27 (1.24) | 38.48 (1.19) | 0,042 |
| Mean blood pressure (mmHg) (mean (SD)) | 70.53 (14.66) | 68.00 (18.77) | 0,595 |
| Blood lactate level (mmol/l)(mean (SD)) | 1.67 (1.02) | 2.68 (3.04) | 0,059 |
| Urea (mmol/l) (mean (SD)) | 4.23 (2.00) | 6.51 (4.23) | 0,003 |
| Serum creatinine (µmol/L) (mean (SD)) | 74.88 (33.61) | 78.58 (50.17) | 0,742 |
| LDH (IU/L) (mean (SD)) | 740.43 (1817.87) | 1491.09 (1974.13) | 0,209 |
| Troponine level (ng/mL) (mean (SD)) | 25.07 (33.13) | 55.66 (28.28) | 0,026 |
| Leucocytes (x103/L) (mean (SD)) | 2018.05 (3090.22) | 349.17 (507.14) | 0,067 |
| Neutrophil (x103/L) (mean (SD)) | 3030.00 (2931.54) | 310.00 (NA) | NA |
| CRS grade upon ICU admission (%) |  |  | 0,09 |
| 0 | 8 (10.4) | 4 (33.3) |  |
| 1 | 25 (32.5) | 3 (25.0) |  |
| 2 | 38 (49.4) | 3 (25.0) |  |
| 3 | 6 (7.8) | 2 (16.7) |  |

**Supplemental Figure S1. Multivariate analysis of factors associated with AKI (including fungal infections)**





LDH > 350 IU/l: OR= 1.23 [1.04 - 1.45], p= 0.019

Extra-renal SOFA score: OR= 1.02 [0.98 - 1.06], p= 0.34

CRS grade ≥ 3: OR= 1.49 [1.08 - 2.06], p= 0.017

Fungal infection: OR= 1.87 [0.98 - 3.57], p= 0.06
